# Is there a preferred IMRT technique for left‐breast irradiation?

**DOI:** 10.1120/jacmp.v16i3.5266

**Published:** 2015-05-08

**Authors:** Marloes Jeulink, Max Dahele, Philip Meijnen, Ben J. Slotman, Wilko F.A.R. Verbakel

**Affiliations:** ^1^ Department of Radiotherapy VU University Medical Center Amsterdam The Netherlands

**Keywords:** breast radiotherapy, IMRT, VMAT, RapidArc, arc therapy

## Abstract

Not all clinics have breath‐hold radiotherapy available for left‐breast irradiation. However intensity‐modulated radiotherapy (IMRT) has also been advocated as a means of lowering heart doses. There is currently no large‐scale, long‐term follow‐up data after breast IMRT and, since dose distributions may differ from classic tangent‐based radiotherapy, caution is needed to avoid unexpected worsening of the late toxicity profile. We compared four IMRT techniques for free‐breathing left‐breast irradiation. Consistent with the aforementioned concerns, our goal in planning was to prioritize organ at risk (OAR) sparing in a way that mimicked tangent‐based radiotherapy. Ten simultaneous integrated boost treatment plans $\left({\text{PTV}}_{\text{elective}}=15\times 2.67\;\text{Gy};{\text{PTV}}_{\text{boost}}=15\times 3.35\;\text{Gy}\right)$ were created using 1) hybrid‐IMRT (H‐IMRT), 2) full IMRT (F‐IMRT), and 3) volumetric‐modulated arc therapy with two partial arcs (2ARC) and 4) six partial arcs (6ARC). Reduction in OAR mean and low dose was prioritized. End‐points included OAR sparing (e.g., heart, left anterior descending artery [LAD+3 mm], lungs, and contralateral breast) and PTV coverage/dose homogeneity. Under these conditions we found the following: 1) H‐IMRT provided the best mean and low dose OAR sparing, ${\text{PTV}}_{\text{elective}}$ coverage $\left(\text{mean}\;V95\%=98\%),{\text{PTV}}_{\text{boost}}\;\text{coverage}\;(V95\%=98\%\right)$, and PTV homogeneity. However, it delivered most intermediate–high dose to the heart, LAD+3 mm and ipsilateral lung; 2) 6ARC had the best intermediate–high dose sparing, followed by F‐IMRT, but this was at the expense of more dose in the contralateral lung and breast and worse PTV coverage (${\text{PTV}}_{\text{elective}}\;\text{mean}\;V95\%=96\%/97\%$ and ${\text{PTV}}_{\text{boost}}\;\text{mean}\;V95\%=91\%/96\%$ for 6ARC/F‐IMRT). When trying to spare mean and low dose to OARs, the preferred IMRT technique for left‐breast irradiation without breath‐hold was H‐IMRT. This is currently the standard solution in our institution for left‐breast radiotherapy under free‐breathing and breath‐hold conditions.

PACS numbers: 87.53kn, 87.53Jw, 87.55.D‐, 87.55.de, 87.55.dk

## I. INTRODUCTION

Cardiac toxicity has been a major concern in left‐breast irradiation.^1^, ^2^ The use of physical techniques such as voluntary deep inspiration breath‐hold (DIBH) to displace the target volume (breast) away from the heart have found favor as a means of protecting the heart.^(3)^ It is clear, however, that for various reasons such techniques are not universally available.^4^, ^5^ In this context, a number of authors have shown that using intensity‐modulated radiotherapy (IMRT) can also reduce heart doses.^6^, ^7^ However there are some uncertainties around the use of full IMRT.

For example: 1) it is generally associated with a larger volume of low dose spread to healthy tissue which has led to concerns about increased risk of second cancers;^(8)^ 2) a linear correlation between heart toxicity and estimated mean dose has been reported,^(2)^ but this does not answer the question of whether a little high dose to the heart (e.g., with tangential radiotherapy) is better or not than more low dose (e.g., with IMRT); and 3) classic tangential breast radiotherapy techniques are characterized by sparing of the nontreated breast and contralateral lung and deliver a variable volume of high‐dose radiation to the heart and ipsilateral lung. If IMRT dose distributions deviate substantially from this, then there is at least a theoretical concern that the late organ‐specific toxicity profile could also differ. Similar concerns have been raised in the use of IMRT for lymphoma, for example.^9^, ^10^ Multiple different hybrid, fixed‐beam, and arc‐based IMRT techniques are now available and, depending on the planning criteria, it is plausible that some may perform better than others. Our aim was to take the typical dose distribution of the classic tangent‐based field arrangement as a benchmark and to compare dosimetric characteristics of four previously described IMRT techniques under conditions of free‐breathing, left‐breast irradiation using a simultaneous integrated boost technique (SIB).^6^, ^7^, ^11^, ^12^, ^13^, ^14^, ^15^ Our primary goal was to investigate their suitability for treatments that stress a reduction in mean and low doses to the heart, lungs, and contralateral breast. Of note is that this general strategy is in line with the recent RTOG 1005 protocol for whole‐breast and SIB irradiation.^(16)^

## II. MATERIALS AND METHODS

The following IMRT techniques were compared: 1) hybrid conventional IMRT, which we currently use routinely in the clinic and which has previously been described in the literature;^(11)^ 2) full conventional IMRT;^(6)^ 3) VMAT^13^, ^14^, ^15^, ^17^, ^18^, ^19^ using RapidArc (Varian Medical Systems, Palo Alto, CA) with two partial arcs, and 4) RapidArc with a previously described technique using six short partial arcs.^(12)^ All techniques are described in detail below in the “Treatment planning” section.

### A. Patients and delineation

CT datasets (General Electric CT LightSpeed 16 (GE Healthcare, Waukesha, WI), slice thickness 2.5 mm, free‐breathing, patients positioned supine on a breast board) of ten patients previously treated for left‐sided breast cancer with breast‐conserving surgery followed by radiotherapy to the breast alone, including a simultaneous integrated boost (SIB) to the tumor bed, were identified for this IMRT planning study. The individual boost volumes were located medial (n=7) or central (n=3) in the breast. These locations were selected because they are closer to heart and contralateral breast and tend to be more challenging for OAR sparing than lateral boosts.

**Figure 1:**
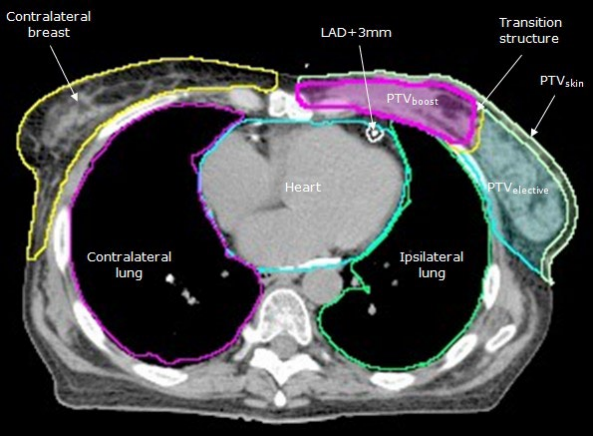
Planning target volume (PTV) and organ‐at‐risk structures (LAD=left anterior descending coronary artery).

Visible breast tissue was delineated, with medial and lateral borders defined by palpation and identified on the planning CT by radiopaque markers placed prior to scanning. The planning target volume (${\text{PTV}}_{\text{breast}}$) was created by expanding the breast with a 5 mm margin. A boost clinical target volume was delineated and expanded by 5 mm to create the boost planning target volume (${\text{PTV}}_{\text{boost}}$). ${\text{PTV}}_{\text{boost}}$ was excluded from ${\text{PTV}}_{\text{breast}}$. A transition structure (TS) of 5 mm between ${\text{PTV}}_{\text{boost}}$ and ${\text{PTV}}_{\text{breast}}$ was also subtracted from ${\text{PTV}}_{\text{breast}}$ and used to facilitate dose falloff between ${\text{PTV}}_{\text{boost}}$ and ${\text{PTV}}_{\text{breast}}$. For optimization, ${\text{PTV}}_{\text{boost}},{\text{PTV}}_{\text{breast}}$, and TS were cropped 5 mm from the skin surface. The skin surrounding the breast tissue that was excluded from ${\text{PTV}}_{\text{boost}},{\text{PTV}}_{\text{breast}}$, and TS was called ${\text{PTV}}_{\text{skin}}$. This structure was used in the inverse optimization process to ensure that the multileaf collimator (MLC) was open around the entire breast contour, including the skin (Fig. 1). In a clinical setting, we plan on a PTV that extends beyond the breast surface to account for breathing motion and setup uncertainty. However, for the purpose of simplicity for this static planning study, PTV outside the breast was not taken into account. A 5 cm wide ring structure at 0 mm outside the PTVs was also used during optimization in order to avoid hotspots outside both PTVs. ${\text{PTV}}_{\text{breast}}$ plus the TS formed ${\text{PTV}}_{\text{elective}}$. Because we are using a SIB technique, we have chosen to report the doses on the boost and elective PTV volumes separately, rather than the combined PTV structure.

During optimization, objectives were set on contralateral breast (CB), heart, left anterior descending artery plus a margin of 3 mm (LAD+3 mm), and ipsilateral and contralateral lung (IL and CL, respectively). The medial border of the contralateral breast was palpated and identified on CT with a radiopaque marker. The heart and LAD were delineated.^(20)^ Both lungs were contoured using autosegmentation. Organ at risk (OAR) and PTV structures were allowed to overlap.

### B. Treatment planning

For each patient, four plans were made using different coplanar IMRT techniques. The prescribed dose (PD) to ${\text{PTV}}_{\text{elective}}$ was 40.05 Gy (15 fractions of 2.67 Gy in three weeks) and the PD to ${\text{PTV}}_{\text{boost}}$ was 50.25 Gy (15 fractions of 3.35 Gy in three weeks). Plans were normalized to provide approximately the same mean dose to ${\text{PTV}}_{\text{boost}}$.. We chose this normalization method instead of, for example, normalizing on a certain PTV coverage, because the PTV dose coverage acceptance criteria may vary between institutions and clinicians. All plans were made using the Eclipse treatment planning system (Varian Medical Systems; dose volume optimizer v.8.9.08 and progressive resolution optimizer v.10.0; continue previous optimization for RA plans, anisotropic analytical calculation algorithm v.8.9.08, with 2.5 mm calculation grid). Six MV photon beams were used for all plans with a maximum dose rate of 600 MU/min.

**Figure 2:**
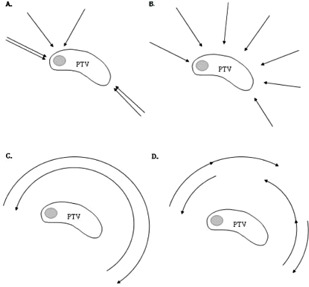
Typical beam/arc arrangements for the various techniques used in this study. The planning target volume (PTV) of the breast is outlined with a medial boost (shaded): (a) Hybrid IMRT (H‐IMRT); (b) Full IMRT (F‐IMRT); (c) 2 Arc RapidArc (2ARC); (d) 6 Arc RapidArc (6ARC).

Typical beam/arc arrangements are illustrated in Fig. 2. H‐IMRT consisted of two tangential open fields, which delivered 85% of the prescribed dose to ${\text{PTV}}_{\text{elective}}$, and four sequentially optimized IMRT fields. Two IMRT fields were tangential and two were directed at the boost area (gantry angles 30° and 330° were suitable in all patients). The IMRT fields were optimized to provide the remaining elective and boost dose, taking into account the dose from the two tangential open fields as a base‐dose plan. MLC was used in the two open fields to block OARs as much as possible without compromising PTV coverage.

Full IMRT (F‐IMRT) consisted of seven coplanar sliding‐window IMRT fields positioned equidistant around the left breast with gantry angles 140°–300°. Collimator angles varied from 0°–10° to avoid possible overlapping tongue and groove effect. The position of X and Y jaws were decided by the planning system according to the corresponding fluence map.

Two arc RapidArc (2ARC) comprised two partial arcs, each irradiating between gantry angles 160° to 290°–305°, with collimator angles of 5°–10°. The fields were based on a conventional open field beam's eye view (BEV) from a classic tangential angle. The position of the X and Y jaws was decided such that most of the breast was within the field for the entire arc, with maximum X field <18 cm.

Six arc RapidArc (6ARC) consisted of six short partial arcs, as described by Tsai et al.,^(12)^ irradiating between gantry angles 305°–355°, 355°–45°, 80°–130°, 130°–80°, 80°–30°, and 355°–305°. For each arc, the jaw opening on the side near the chest wall was minimized to reduce the exposure to the left lung and the right breast, resulting in smaller jaw openings than those used in the 2ARC plans.

Individual planning objectives were used for each patient and adjusted according to patient anatomy. These objectives could vary between patients, but to avoid bias, for each individual patient the same optimization objectives were used for the full IMRT, 2ARC, and 6ARC plans. For the H‐IMRT technique, different optimization objectives were used because 85% of the dose was delivered with tangential fields. Limiting low‐dose irradiation was considered clinically important and it can also make a significant contribution to mean OAR doses. Low (e.g., V5), intermediate (e.g., V20), and high (e.g., V40) OAR doses were constrained for heart, IL, and CL. Additional objectives were set on CB, LAD+3 mm, ${\text{PTV}}_{\text{skin}},{\text{PTV}}_{\text{boost}},{\text{PTV}}_{\text{breast}}$, and TS. During optimization, OAR objectives were interactively adjusted to keep them below the DVH line, up to the point where ${\text{PTV}}_{\text{elective}}$ and ${\text{PTV}}_{\text{boost}}$ coverage started to be compromised. Priorities for PTV objectives were similar to those for the ipsilateral OAR low‐dose objectives, whereas priorities for contralateral OAR and high‐dose objectives in ipsilateral OAR were set approximately 10% lower.

### C. Analysis

In the literature, a wide variation of OAR doses is reported and the optimal OAR dose profile in breast radiotherapy remains uncertain. Therefore, we choose to report multiple parameters for evaluation:

1. PTV coverage defined as the percentage of ${\text{PTV}}_{\text{elective}}$ and ${\text{PTV}}_{\text{boost}}$ receiving ≥ 95% of the PD (V95%). V95 ≥ 95% was considered clinically acceptable.
2. PTV dose homogeneity defined as ${\text{PTV}}_{\text{elective}}$ and ${\text{PTV}}_{\text{boost}}$ standard deviation (SD) and ${\text{PTV}}_{\text{elective}}$ and ${\text{PTV}}_{\text{boost}}$ V107%.
3. OAR doses for heart ($D_{\text{mean}}$, V5, V10, V20, and V30), LAD+3 mm (V20 and V30), IL ($D_{\text{mean}}$, V5, V20, V30), CL ($D_{\text{mean}}$) and CB ($D_{\text{mean}}$, V5, V10).
4. For total body exposure, V5 of the body contour and the number of monitor units (MU) were evaluated.

Statistical analyses were performed by comparing H‐IMRT respectively to F‐IMRT, 2ARC, and 6ARC using the paired nonparametric Wilcoxon signed‐rank test (SPSS statistical software (v.21, SPSS Inc.; IBM Corporation, NY)). A p‐value of ≤ 0.05 was considered significant.

## III. RESULTS

Mean ±SD volumes of ${\text{PTV}}_{\text{elective}}$ and ${\text{PTV}}_{\text{boost}}$ were 728±554 and 83±52 cc, respectively. Mean ±SD volumes of heart, LAD+3 mm, CB, CL, and IL were 692±167, 8±2, 842±435, 1558±243, 1355±208 cc, respectively. Pooled data from all ten patients are presented in Table 1. The most favorable technique for each parameter is noted.

**Table 1:** Comparison of planning target volume (PTV) and organ‐at‐risk (OAR) dosimetry (n=10).

	*H‐IMRT*		*F‐IMRT*			*2 Arc*			*6 Arc*		
	*Mean*	*STD*	*Mean*	*STD*	*p*	*Mean*	*STD*	*p*	*Mean*	*STD*	*p*
${\text{PTV}}_{\text{elective}}$ mean dose (%)	101.8	0.5	102.4	0.3	0.02	101.2^a^	2.6	0.44	102.3	0.3	0.04
${\text{PTV}}_{\text{elective}}$ STD (%)	3.5^a^	0.6	3.7	0.4	0.31	4.1	0.8	0.04	3.7	0.3	0.44
${\text{PTV}}_{\text{elective}}$ V95% (%)	98.0^a^	0.7	96.5	0.7	0.01	91.9	3.7	0.01	96.0	1.3	0.01
${\text{PTV}}_{\text{elective}}$ V107% (%)	7.4^a^	3.5	11.0	3.5	0.03	12.5	5.5	0.02	12.0	3.7	0.05
${\text{PTV}}_{\text{boost}}$ mean dose (%)	100.1	0.9	100.0^a^	0.8	0.65	100.6	1.2	0.33	100.3	0.9	0.24
${\text{PTV}}_{\text{boost}}$ STD (%)	2.3^a^	0.4	2.7	0.7	0.11	3.9	1.0	0.01	4.2	2.1	0.01
${\text{PTV}}_{\text{boost}}$ V95% (%)	97.9^a^	1.7	95.9	3.0	0.02	91.2	6.0	0.01	90.9	7.4	0.01
${\text{PTV}}_{\text{boost}}$ V107% (%)	0.0^a^	0.0	0.5	0.7	0.04	3.6	4.3	0.01	2.8	3.5	0.01
Heart mean dose (Gy)	3.8^a^	1.8	4.8	1.9	0.09	5.0	1.5	0.03	3.9	1.9	0.96
Heart V5 (%)	23.0^a^	16.1	28.1	19.9	0.51	31.8	17.7	0.17	25.5	23.6	0.96
Heart V10 (%)	6.4	8.8	8.8	9.9	0.72	9.3	7.3	0.09	5.7^a^	6.8	0.31
Heart V20 (%)	2.9	2.7	1.6	2.0	0.07	1.4	1.7	0.01	0.5^a^	0.9	0.01
Heart V30 (%)	1.9	1.9	0.2	0.3	0.01	0.2	0.4	0.01	0.0^a^	0.1	0.01
LAD+3 mm V20 (cc)	3.0	2.8	1.6	1.7	0.05	1.7	1.7	0.03	1.0^a^	1.5	0.01
LAD+3 mm V30 (cc)	2.4	2.6	0.1^a^	0.3	0.02	0.3	0.8	0.02	0.1^a^	0.4	0.02
IL mean dose (Gy)	6.5	2.3	6.1	1.3	0.45	7.1	1.5	0.17	5.1^a^	83.0	0.04
IL V5 (%)	26.7	9.3	34.0	12.6	0.02	40.4	11.4	0.01	24.7^a^	4.3	0.24
IL V20 (%)	11.7	5.5	7.0	2.0	0.01	9.1	3.4	0.07	6.0^a^	2.2	0.01
IL V30 (%)	8.7	4.9	2.6	1.3	0.01	2.8	1.9	0.01	1.7^a^	1.2	0.01
CL mean dose (Gy)	0.5^a^	0.3	1.2	0.3	0.01	1.7	3.8	0.01	1.8	0.7	0.01
CB mean dose (Gy)	0.6^a^	0.3	1.6	0.2	0.01	2.4	0.4	0.01	2.4	0.4	0.01
CB V5 (%)	1.3^a^	1.4	4.1	1.6	0.01	6.7	2.3	0.01	6.8	2.7	0.01
CB V10 (%)	0.4	0.7	0.2	0.3	0.67	0.1^a^	0.3	0.07	0.3	0.6	0.89
Body V5 (cc)	2416.0^a^	1061.9	3259.3	1414.6	0.01	4227.4	2097.3	0.01	3445.3	1487.5	0.01
MU	698.1^a^	98.7	1546.9	320.0	0.01	729.5	62.9	0.20	902.8	86.6	0.01

^a^ The most favorable numerical result.

${\text{PTV}}_{\text{elective}}=\text{elective planned target volume}$; SD=standard deviation; ${\text{PTV}}_{\text{boost}}=\text{boost planned target volume}$; IL=ipsilateral lung; H−IMRT=hybrid intensity−modulated radiotherapy; CL=contralateral lung; F−IMRT=full intensity−modulated radiotherapy; CB=contralateral breast; 2 ARC=volumetric−modulated arc therapy (RapidArc) using two partial arcs; LAD=left anterior descending artery; 6 ARC=volumetric−modulated arc therapy (RapidArc) using 6 short partial arcs; MU=monitor units.

Only H‐IMRT and F‐IMRT achieved ${\text{PTV}}_{\text{elective}}$ and ${\text{PTV}}_{\text{boost}}$ coverage of V95% >95%. 2ARC was associated with considerably lower ${\text{PTV}}_{\text{elective}}$ coverage than other techniques, although its ${\text{PTV}}_{\text{boost}}$ coverage was comparable to 6ARC. Dose homogeneity was best for H‐IMRT.

**Figure 3:**
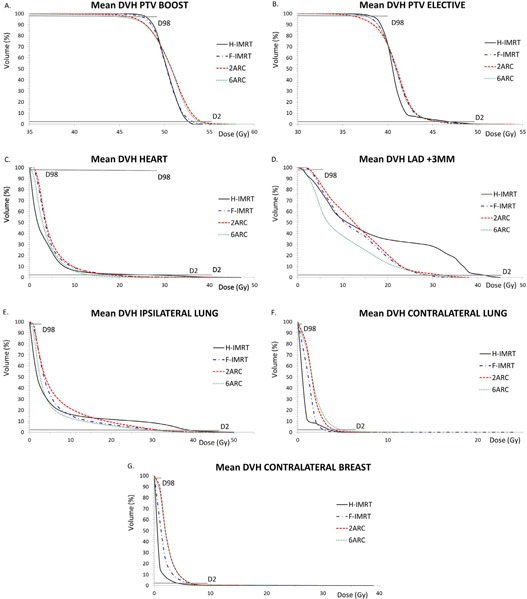
Mean dose‐volume histograms for planning target volume (PTV) and organs at risk averaged over all patients: (a) ${\text{PTV}}_{\text{boost}}$; (b) ${\text{PTV}}_{\text{elective}}$; (c) heart; (d) LAD+3 mm; (e) ipsilateral lung; (f) contralateral lung; (g) contralateral breast.

Absolute average mean dose for heart (3.8±1.8 Gy), CB (0.6±0.3 Gy), and CL (0.5±0.3 Gy), and V5 for heart (23.0%±16.1%) and CB (1.3±1.4 Gy) were smallest with H‐IMRT. Despite the low mean heart dose, H‐IMRT delivered the highest V20 (2.9%±2.7%) and V30 (1.9%±1.9%) to the heart, and the highest V20 (3.0±2.8 cc) and V30 (2.4±2.6 cc) to LAD+3 mm. This emphasizes the important contribution of larger volumes of low doses (e.g., V5 and V10) to the mean OAR dose. Ipsilateral lung doses (mean and V5/20/30) were lowest with 6ARC ($D_{\text{mean}}$ 5.1 Gy, V5=24.7%,V20=6.0%,V30=1.7%), which also achieved the smallest volumes of intermediate–high dose (V10/20/30) in the heart (V20=0.5%) and LAD+3 mm (V20=1 cc). In some cases, when the heart and LAD doses were high with H‐IMRT, 6ARC could reduce these. For example, in one patient, H‐IMRT delivered ≥ 20 Gy to 6.1 cc of LAD+3 mm and 6ARC reduced this to 0.5 cc. Figure 3 shows the DVH curves for a typical patient.

For seven out of ten cases, H‐IMRT used the least MU. F‐IMRT used the most (more than double H‐IMRT) for all cases. 6ARC used more MU than 2ARC, but although both used fewer MU than F‐IMRT, the V5 of the body was higher with the RapidArc techniques than with F‐IMRT. The body V5 was lowest with H‐IMRT.

In eight patients, 6ARC provided better ${\text{PTV}}_{\text{boost}}$ and ${\text{PTV}}_{\text{elective}}$ coverage than 2ARC, but this does not show in the results for ${\text{PTV}}_{\text{boost}}$ due to two outliers. This is because in the two patients with the most medially located boost, 6ARC achieved substantially less ${\text{PTV}}_{\text{boost}}$ coverage than 2ARC due to the fact that the X jaw was closed on the ipsilateral lung and blocked ${\text{PTV}}_{\text{boost}}$ in important tangential directions. While, in general, this allowed for good ipsilateral lung sparing in these two cases, it did not allow the ${\text{PTV}}_{\text{boost}}$ to be within the beam long enough to achieve good coverage with the 6ARC technique.

To better understand the relation between PTV coverage and low dose objectives, we removed these on CL and CB in one of the 2ARC plans. This allowed excellent boost and elective PTV coverage to be achieved, (${\text{PTV}}_{\text{boost}}$ V95%=99.0%, ${\text{PTV}}_{\text{elective}}$ V95%=97.3%), at the expense of CL (V5=22.5%) and (CB V5=86.7%), compared to the plan with low‐dose objectives on CL and CB (${\text{PTV}}_{\text{boost}}$ V95%=88.8%, ${\text{PTV}}_{\text{elective}}$ V95%=94.5%,CL V5=0.3%,CB V5=6.8%).

## IV. DISCUSSION

Under the boundary condition of limiting mean and low dose to lung, heart, and contralateral breast, we found that H‐IMRT achieved best OAR sparing and acceptable PTV coverage for free‐breathing, left‐breast IMRT irradiation. Under consistent planning conditions, the results illustrate differences between IMRT techniques, and suggest that the preferred OAR dose profile will influence the choice of IMRT technique. We have also illustrated the extent to which parameters such as VMAT arc angles can influence the performance of a given technique.

Most RapidArc planning studies describe a technique that uses two partial arcs or one single arc for the treatment of breast cancer.^13^, ^14^, ^15^, ^17^ However this may be suboptimal when constraining OAR low dose is considered a priority. Under such conditions, our 2ARC technique failed to provide acceptable PTV coverage, showed least low‐dose sparing, and less high‐dose sparing than both F‐IMRT and 6ARC. While the increased volume of low‐dose with VMAT (and conventional IMRT) is recognized, it is not clear that this is always constrained during planning.^(19)^ Although RapidArc is capable of modulating MLC position and dose rate while the gantry is rotating, it is programmed to rotate with maximum gantry speed. In the case of breast irradiation, it is possible that it may not be able to deliver sufficient modulated dose from optimal tangential directions, impairing PTV coverage, especially when trying to simultaneously limit the OAR dose. In addition, the dosimetric differences between the 2ARC and 6ARC techniques show that the arrangement of the arcs can significantly influence the final quality of the treatment plan.

It is clear that an incomplete understanding of the radiobiology of breast radiotherapy and its toxicity is hampering the design and evaluation of treatment techniques.^(21)^ Important issues that remain unresolved include, for example: 1) the optimal trade‐off between low and high heart doses for treatment planning and the effect of volume; 2) which part(s) of the heart are most important to spare (for instance, Darby et al.^(2)^ found that the mean dose to the LAD was not a significant predictor after taking into account the heart dose); and 3) whether the interaction between radiation and other risk factors for late toxicity such as chemotherapy are also dose‐volume dependent.

Recently, several groups have compared VMAT with IMRT and tangential breast irradiation techniques;^17^, ^18^, ^19^ however, the present study differs from these, in particular because we aimed to optimize all techniques to deliver small volumes of low dose to heart and lung, and we investigated two different VMAT techniques. Both our multiple short arc VMAT and the more conventional two‐arc VMAT managed to achieve lower mean heart doses than earlier reported, although sometimes the price to be paid was a lower PTV coverage. A wide variety of plan results can be achieved with VMAT, depending for example on the number and length of arcs and optimization objectives.

The sample size is typical for a planning study.^11^, ^12^, ^13^, ^14^ Although our table presents average data, a more individualized approach may be required when deciding the best IMRT technique. F‐IMRT and 6ARC were best at sparing LAD+3 mm at the expense of larger volumes of low dose in other OAR. However, in four out of ten patients, H‐IMRT managed to spare the LAD+3 mm, as well as F‐IMRT and 6ARC, with LAD+3 mm V30 of 0.0 cc in all cases. In these cases, the favorable anatomy allowed blocking of the LAD+3 mm without affecting PTV coverage. Awareness of such characteristics is important to help with the selection of the most appropriate alternative when the first‐choice solution (in this case H‐IMRT) does not deliver the desired plan. We expect that a drawback of the H‐IMRT technique, namely high dose in the heart and LAD+3 mm, could be reduced in many patients by combining it with deep inspiration breath‐hold (DIBH) to increase the PTV–OAR distance.^(3)^ H‐IMRT can also be delivered using high‐dose‐rate flattening filter‐free beams,^(22)^ which can help reduce the beam delivery time and could be useful for a DIBH technique. For most patients in our clinic we use the H‐IMRT technique delivering 85% of the dose with the open tangential fields in close analogy with our H‐IMRT technique for locally advanced lung cancer.^(23)^ It retains the advantage of tangential delivery while providing sufficient room to modulate and conform different dose levels. Selected patients may benefit from a more individualized open tangential field to IMRT dose ratio.

## V. CONCLUSIONS

This study illustrates that, for the OAR choices described here, free‐breathing, left‐breast IMRT planning could generally start with H‐IMRT, followed by 6ARC VMAT if it is thought that H‐IMRT cannot spare the LAD, heart, and ipsilateral lung sufficiently from high doses.

## ACKNOWLEDGMENTS

We gratefully acknowledge D. Rietveld and K. Spruijt (contouring).

## Supporting information

Supplementary Material
