# Supplementary figures and images for: Is there a preferred IMRT technique for left‐breast irradiation?
Source: J Appl Clin Med Phys. 2015 May 8;16(3):197–205. doi: 10.1120/jacmp.v16i3.5266 (PMC5690145; doi:10.1120/jacmp.v16i3.5266)

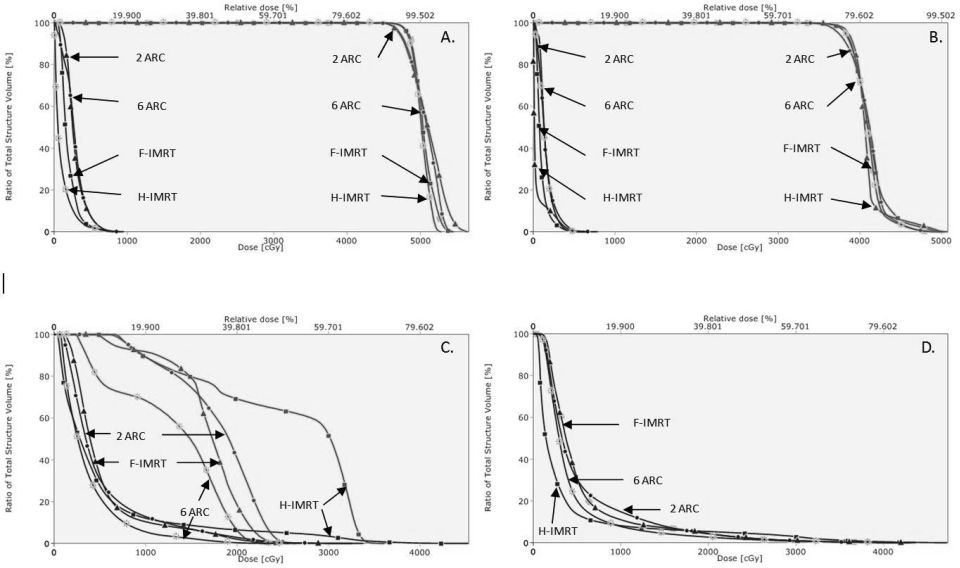

Supplement: Supplementary file 1 — Supplementary Material [file ACM2-16-197-s001.jpg]
